# Diphenyleneiodonium chloride (DPIC) displays broad-spectrum bactericidal activity

**DOI:** 10.1038/s41598-017-11575-5

**Published:** 2017-09-14

**Authors:** Manitosh Pandey, Alok Kumar Singh, Ritesh Thakare, Sakshi Talwar, Pratiksha Karaulia, Arunava Dasgupta, Sidharth Chopra, Amit Kumar Pandey

**Affiliations:** 1 Mycobacterial Pathogenesis Laboratory, Vaccine and Infectious Disease Research Centre (VIDRC), Translational Health Science and Technology Institute (THSTI), NCR Biotech Science Cluster, Faridabad-Gurugram Expressway, Faridabad, 121001 Haryana India; 2 Division of Microbiology, CSIR-Central Drug Research Institute, Sector-10, Janakipuram Extension, Sitapur Road, Lucknow, 226031 Uttar Pradesh India

## Abstract

Indiscriminate use of antibiotics globally has lead to an increase in emergence of drug-resistant pathogens under both nosocomial, as well as more worryingly, in community setting as well. Further, a decrease in the corporate interest and financial commitment has exerted increasing pressure on a rapidly dwindling antimicrobial drug discovery and developmental program. In this context, we have screened the Library of Pharmacologically Active Compounds (LOPAC, Sigma) against *Staphylococcus aureus* and *Mycobacterium tuberculosis* to identify potent novel antimicrobial molecules amongst non-antibiotic molecules. Microplate-based whole cell growth assay was performed to analyze the antimicrobial potency of the compounds against *Staphylococcus aureus* and *Mycobacterium tuberculosis*. We identified diphenyleneiodonium chloride, a potent inhibitor of NADH/NADPH oxidase, as a broad-spectrum antibiotic potently active against drug resistant strains of *Staphylococcus aureus* and *Mycobacterium tuberculosis*. Intriguingly, the diphenyleneiodonium chloride was also very effective against slow-growing non-replicating Mtb persisters. FIC index demonstrated a strongly synergistic interaction between diphenyleneiodonium chloride and Rifampicin while it did not interact with INH. The antimicrobial property of the diphenyleneiodonium chloride was further validated *in vivo* murine neutropenic thigh *S. aureus* infection model. Taken together, these findings suggest that Diphenyleneiodonium chloride can be potentially repurposed for the treatment of tuberculosis and staphylococcal infections.

## Introduction

Antimicrobial resistance (AMR) is one of the greatest public health challenges being faced by global healthcare systems. AMR not only threatens the effective treatment of currently treatable infections but also negates the advancements made in the field of medical sciences including surgery and cancer immunotherapy, with quantifiable impacts on global healthcare budgets. AMR is widespread, both in the nosocomial as well as community setting and World Health Organization (WHO) has recently released a priority pathogen list against which drugs and diagnostics are urgently required^1^. This dovetails nicely with multiple calls by the Infectious Diseases Society of America (IDSA) and multiple national and international governmental organizations worldwide for augmenting the severely anemic drug discovery pipeline urgently^2,3^.

The impact of drug-resistance is even more severely felt in the control of chronic infections. Amongst the WHO priority two pathogens, *Staphylococcus aureus* (SA) is a commensal and a deadly human pathogen, causing infections ranging from skin and soft tissue infections to bacteremia, infective endocarditis and device related infections^4^. With the emergence of methicillin and vancomycin resistance in *S. aureus*, there is an urgent need to discover novel broad-spectrum antibiotics which will help save millions of life affected by these deadly but otherwise treatable pathogens^5,6^.

In a similar vein, tuberculosis (TB), a chronic infection caused by *M. tuberculosis* (Mtb), is severely impacted by the rise of AMR. During the latent symptomless form of the infection, Mtb persists inside the host for decades, with majority of the patients acting as carriers and roughly one-tenth develop active disease^7,8^. The currently approved treatment (DOTS) consists of Isoniazid (INH), Rifampicin (RIF), Ethambutol (ETB) and Pyrazinamide (PYR) for 2 months followed by INH and RIF for 4 months^9,10^. The treatment regimen could extend from 6 months to 2 years depending on the site of infection as well as the drug-resistance status of the infecting strain^11^. Patient non-compliance is one of the primary causes for generation of multi-drug (MDR) and extremely drug-resistant (XDR) strains, thus further complicating treatment of tuberculosis^12^.

A sub-population of slow-growing non-replicating persisters (NRP) is believed to be the major contributors for the long-term persistence of *S. aureus* and Mtb inside the host^13,14^. These NRPs are refractory to most anti-tubercular drugs and require prolonged time and increased dosage for their clearance^15,16^. Thus, the need of the hour is to identify novel hit compounds which equi-potently target both replicative and the NRP, effecting a significant reduction in treatment duration with a concomitant increase in treatment efficacy.

Keeping these requirements in mind, we screened the Library of Pharmacologically Active Compounds (LOPAC) library consisting of 1280 structurally diverse small molecules and identified Diphenyleneiodonium chloride (DPIC), a known NADH/NADPH oxidase inhibitor as possessing potent antimicrobial activity against Mtb and *S. aureus*. Interestingly, DPIC exerted potent anti-mycobacterial activity against replicative, non-replicative Mtb as well as intracellular Mtb inside the mouse bone marrow derived macrophages. DPIC synergized with RIF while it had no interaction with INH. Finally, DPIC was tested for its antimicrobial activity *in vivo* in the murine neutropenic thigh *S. aureus* infection model where it performed a comparable reduction in bacterial burden to vancomycin that too at 1/25 dosage.

## Results and Discussion

### DPIC is highly potent against *Mycobacterium tuberculosis* and *Staphylococcus aureus*

To identify non-antibiotic inhibitors exhibiting potent antimicrobial activity against Mtb and *S. aureus*, we screened the LOPAC library consisting of 1280 pharmacologically active compounds and identified Diphenyleneiodonium chloride (DPIC), a potent inhibitor of NAPD/NADPH oxidase, with an MIC of 0.03 mg/L against *M*. *bovis* BCG and 1 mg/L against SA (Table 1 and Supplementary Figure 1). In order to identify its antimicrobial spectrum, DPIC was screened against an expanded ESKAPE panel consisting of well defined and characterized clinical *S. aureus* strains and clinical drug-resistant Mtb strains. As can be seen in Table 1, DPIC was equi-potently effective against drug-resistant clinical isolates of *S. aureus* as compared to *S. aureus* ATCC 29213 (MIC 0.5–1 mg/L). Similar pattern of efficacy was demonstrated against clinical drug-resistant strains of Mtb with an MIC comparable to that of Mtb H_37_Rv ATCC 27294 (0.03 mg/L). In contrast, DPIC lacked any significant potency against gram-negative bacteria with MIC ranging from 4–32 mg/L. Our data correlates very nicely with earlier publication by Altaf *et al*. in 2010 where they also reported potent antimicrobial activity of DPIC against H_37_Rv (MIC of 0.39 µM or 0.12 mg/L)^17^. All together, DPIC was equally potent against drug sensitive as well as drug resistant mycobacterial and *S. aureus*

**Table 1:** MIC values of DPIC against a multi-organism clinical strain panel.

Strains		Antibiotics resistant to	Molecular details of strains	MIC of DPIC (mg/L)
***M. tuberculosis***	H37Rv ATCC 27294	None	Type strain	0.03
	INH resistant ATCC 35822	INH		0.03
	RIF resistant ATCC 35838	RIF		0.03
	ETB resistant ATCC 35837	ETB		0.03
	STR resistant ATCC 35820	STR		0.03
**MSSA**	SA 29213	None	Type strain	1
**MRSA**	NR 119	Methicillin, Ceftriaxone, Meropenem, Gentamycin and Linezolid	• Positive for mec (subtype IV) • G2576T mutation in domain V in one or more 23S rRNA genes	2
	NR 100	Methicillin, Ceftriaxone, Meropenem	• Resistant to tetracycline • Positive for mec (subtype I) • Large variety of virulence factors	1
	NR 10129	Methicillin, Ceftriaxone, Meropenem	• Also known as TCH60	2
	NR 10198	Methicillin, Ceftriaxone, Meropenem	• Community acquired-MRSA • Pulse-field gel electrophoresis (PFGE) typed as USA100 • Negative for the *Panton-Valentine leucocidin* (PVL) virulence factor. • Contains staphylococcal chromosome cassette mec type II.	2
	NR 10192	Methicillin, Ceftriaxone, Meropenem	• Community acquired-MRSA • Pulse-field gel electrophoresis (PFGE) typed not as USA100-1100 • Negative for the *Panton-Valentine leucocidin* (PVL) virulence factor. • Contains staphylococcal chromosome cassette mec type II.	2
	NR 10191	Methicillin, Ceftriaxone, Meropenem	• Community acquired-MRSA • Pulse-field gel electrophoresis (PFGE) typed as USA600 • Negative for the *Panton-Valentine leucocidin* (PVL) virulence factor. • Contains staphylococcal chromosome cassette mec type II.	2
	NR 10193	Methicillin, Ceftriaxone, Meropenem	• Community acquired-MRSA • Negative for the *Panton-Valentine leucocidin* (PVL) virulence factor. • Contains staphylococcal chromosome cassette mec type II.	1
	NR 10186	Methicillin, Ceftriaxone, Meropenem	• Community acquired-MRSA • Pulse-field gel electrophoresis (PFGE) typed as USA300 • Positive for the *Panton-Valentine leucocidin* (PVL) virulence factor. • Contains staphylococcal chromosome cassette mec type IV	0.5
	NR 10194	Methicillin, Ceftriaxone	• Community acquired-MRSA • Positive for the *Panton-Valentine leucocidin* (PVL) virulence factor. • Contains staphylococcal chromosome cassette mec type V	1
**VRSA**	VRS1	Methicillin, Ceftriaxone, Meropenem, Gentamycin, Vancomycin, Teicoplanin	• Positive for *mec* (subtype II) and *vanA* • Negative for *vanB, vanC1, vanC2, vanD, vanE, PVL* and arginine catabolic mobile element (ACME) • Pulsed-field type USA100	1
	VRS4	Methicillin, Ceftriaxone, Meropenem, Vancomycin and Teicoplanin	• Positive for mec (subtype II) and *vanA* • Negative for *vanB, vanC1, vanC2, vanD, vanE, PVL* and arginine catabolic mobile element (ACME) • Pulsed-field type USA100	2
	VRS12	Methicillin, Ceftriaxone, Meropenem, Vancomycin and Teicoplanin	• NA*	2
**Gram-negative bacteria**	*Escherichia coli* ATCC 25922	None	Type strain	4
	*Klebsiella pneumoniae* BAA-1705	Carbapenem-resistant (Imipenem and Ertapenem)	Type strain	16–32
	*Acinetobacter baumannii* BAA-1605	Ceftazidime, Gentamicin, Ticarcillin, Piperacillin, Aztreonam, Cefepime, Ciprofloxacin, Imipenem and Meropemem	Type strain	4
	*Pseudomonas aeruginosa* ATCC 25923	None	Type strain	4–8

NA: Not available

clinical strains while significantly less potent against gram-negative bacteria, thus indicating a potentially new mechanism of action and lack of cross-resistance with existing drugs.

### Bacterial killing kinetics with DPIC

**Figure 1:**
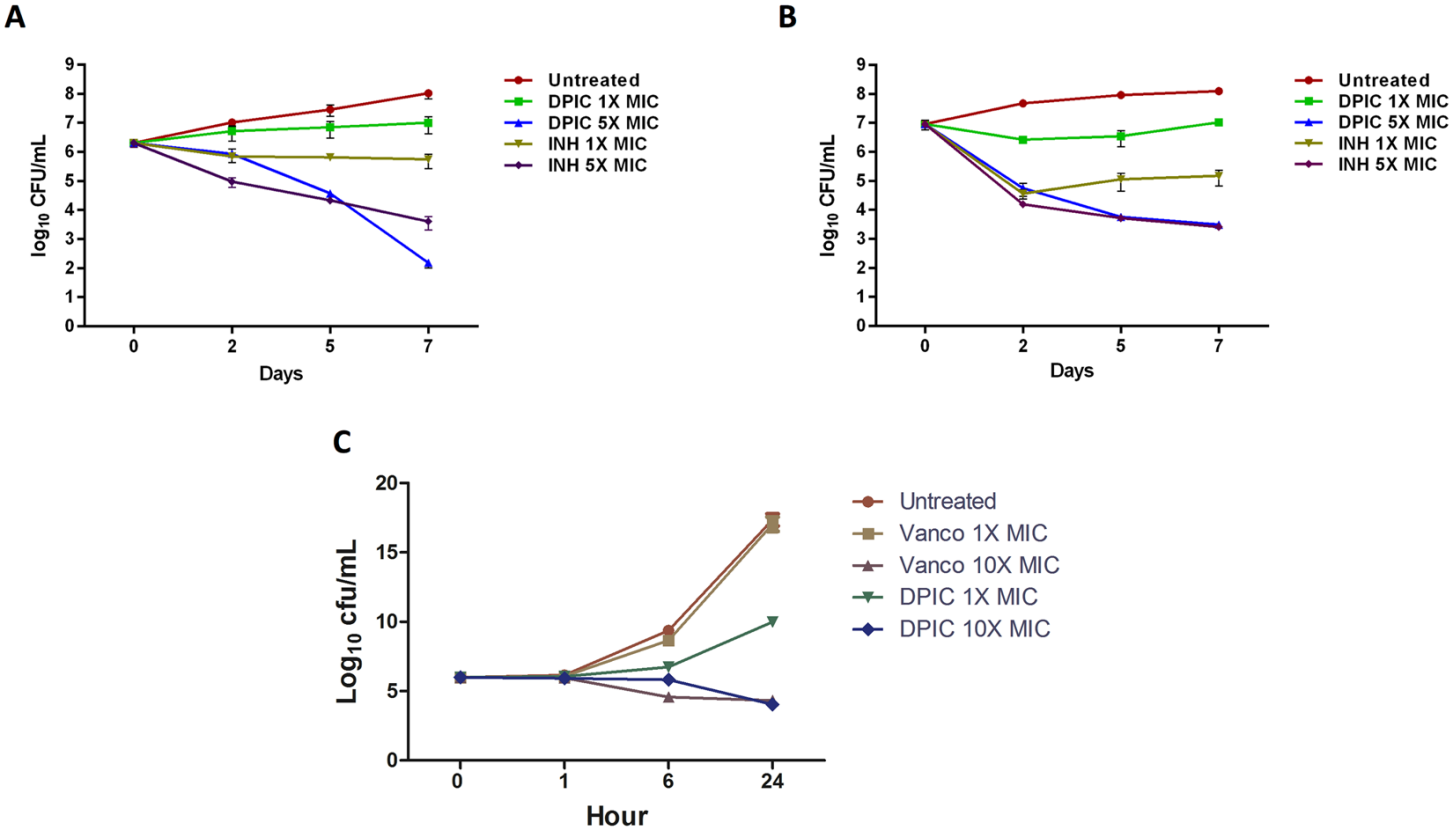
DPI demonstrated a dose dependent bactericidal activity against *M. tuberculosis* and *S. aureus*. Killing curve for various drugs depicting concentration dependent killing in *M. tuberculosis* (**A**) replicative media, (**B**) non-replicative media, (**C**) in S. aureus, bacterial enumeration was done by CFU plating at different time points on 7H11 + OADC plates and MHA plates for *M.tb* and *S. aureus* respectively.

After determining the antimicrobial potential of DPIC, we next assessed the bacterial killing kinetics various concentrations of DPIC with INH and vancomycin as controls against Mtb H_37_Rv ATCC 27294 and *S. aureus* under replication permissive conditions respectively. As depicted in Fig. 1A, DPIC exhibits very modest killing kinetics at 1X MIC while causing a ~4.3 log_10_ cfu reduction at 5X MIC in 7 days as compared to no drug control. In contrast, INH exhibited a ~2.3 log_10_ cfu killing at 1X MIC and ~4.3 log_10_ cfu reduction at 5X MIC. Interestingly, there is no re-growth of Mtb bacilli after 96 h at 5X MIC of DPIC, indicating potent concentration dependent bactericidal nature. Against *S. aureus*, DPIC exhibited similar pattern in reduction in cfu with a ~13 log_10_ cfu reduction at 10X MIC which is comparable to vancomycin as seen in Fig. 1C. Thus, DPIC exhibits concentration dependent bactericidal activity against both *S. aureus* and Mtb.

Under replication non-permissive conditions^18^, DPIC reduced ~6 log_10_ cfu at 5X MIC in 7 days but was modest at 1X MIC. In contrast, INH was only able to reduce cfu by ~4 log_10_, thus indicating the better potential of DPIC to eradicate NRP cells when compared to INH (Fig. 1B). Most of the available antibiotics against Mtb target growing bacteria except PZA. PZA is a prodrug that specifically targets the slow-growing non-replicating persisters and its inclusion in the treatment results in shortening of the tuberculosis therapy. Despite all of the above advantages, unlike DPIC the anti microbial activity of PZA is pH dependent and shows activity only in an acidic environment^19^ (Supplementary Table 1). Addition of DPIC as an adjunct therapy exhibits the potential to be very effective as there is a greater chance to eliminate NRP cells. Moreover, DPIC in combination with PZA would potentially drastically reduce the evolution of drug resistance bacteria among the NRP population that otherwise could spontaneously become refractory to one or more drugs.

### DPIC effectively inhibits Mtb replication inside mouse bone marrow-derived macrophages (mBMDM)

**Figure 2:**
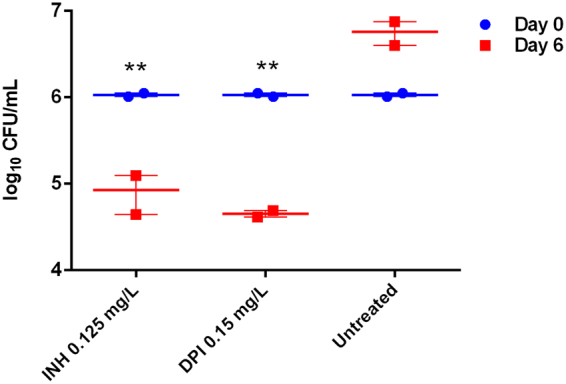
DPIC inhibits the intracellular replication of *M. tuberculosis* inside mouse Bone marrow-derived macrophages. Survival of *M. tuberculosis* in BMDM in the presence of INH and DPIC, drug treatment was done four hours post infection, and were replenished on day 4. Bacterial enumeration was done by lysing the cells with 0.01% triton X-100 and CFU plating on 7H11 + OADC plates.

Since Mtb is an obligate intracellular pathogen, we further wanted to check the anti-microbial efficacy of DPIC against the pathogen replicating inside the cell. The ability of DPIC to restrict intra-cellular growth of Mtb was estimated by comparing CFU six days post infection between DPIC treated and untreated mBMDMs. Before performing the experiment, the cytotoxic effect of DPIC against the eukaryotic cells was evaluated. The CC_50_ of DPIC against Vero cells was found to be 2 mg/L, thus the selectivity index (CC_50_/MIC) was calculated to be 66.67 for Mtb H_37_Rv (Table 2). As can be seen in Fig. 2, DPIC at 5X MIC causes a reduction of ~1.5 log_10_ cfu which is quite comparable to reduction caused by INH ~1 log_10_ cfu as compared to no drug control (P < 0.005). The growth reduction is similar to what others have reported using different first-line antimycobacterial drugs currently in use ref.^20^. Mtb being an intracellular pathogen, the most challenging bottleneck of any anti-infective has been its ability to access its potential target. This requires the drug to breach several barriers namely, host cell membrane, phagosomal membrane and finally the lipid rich cell wall of Mtb. Taken together, DPIC is potently active *ex-vivo* in reducing Mtb growth, thus potentially decreasing the infection relapse rate.

**Table 2:** Cyto-toxicity profile against Vero cells and Selectivity index of DPIC.

**Strain**	Mtb H37Rv	*S. aureus*
Selectivity index	66.67	4

### DPIC synergizes with front-line anti-TB drugs

Anti-tuberculosis therapy involves a combination of multiple drugs administered for long and varied duration. According to the WHO guidelines, any new drug approved for use against tuberculosis will have to be administered as a combination therapy. In order to determine whether DPIC exhibits any interaction with front-line anti-TB drugs, we calculated the FIC index of DPIC in the presence of RIF and INH. As can be seen in Table 3, DPIC demonstrated an FIC index of 0.74 with INH suggesting no interaction while with RIF, the FIC index was 0.28, thus indicating synergistic effect. We validated the above findings by demonstrating similar levels of bactericidal activity at four fold less (0.25x MIC = 7.5 mg/L) RIF concentration (~2 log_10_ reduction in CFU) when administered in combination with 32 fold less (0.03x MIC = 1.95 mg/L) of DPIC as compared to RIF alone at 1x MIC (0.03 mg/L) (Supplementary Figure 2)

**Table 3:** Drug interaction studies of DPIC with front-line Anti-TB drugs.

Drugs in combination	MIC (mg/L)	FIC index	Indication
DPI + RIF	(D) 0.0075 + (R) 0.0019	0.281	Synergy
DPI + INH	(D) 0.015 + (I) 0.031	0.74	No interaction

Collectively, DPIC exhibits the potential to possibly reduce the dosage associated toxicity with RIF.

This is very important since RIF, being a potent inducer of hepatic cytochrome P450, decreases the half-life of several co-administered drugs as well as induces hepatotoxicity^21^. DPIC as an adjunct therapy against tuberculosis could significantly reduce the RIF dosage, alleviating RIF-mediated side effects. Further, evaluating the FIC index of DPIC with respect to other 1^st^ and 2^nd^ line anti tuberculosis drug could help design a better regimen against both drug sensitive as well as drug resistant Mtb infection.

### DPIC effectively inhibits *S. aureus* replication in a murine infection model

**Figure 3:**
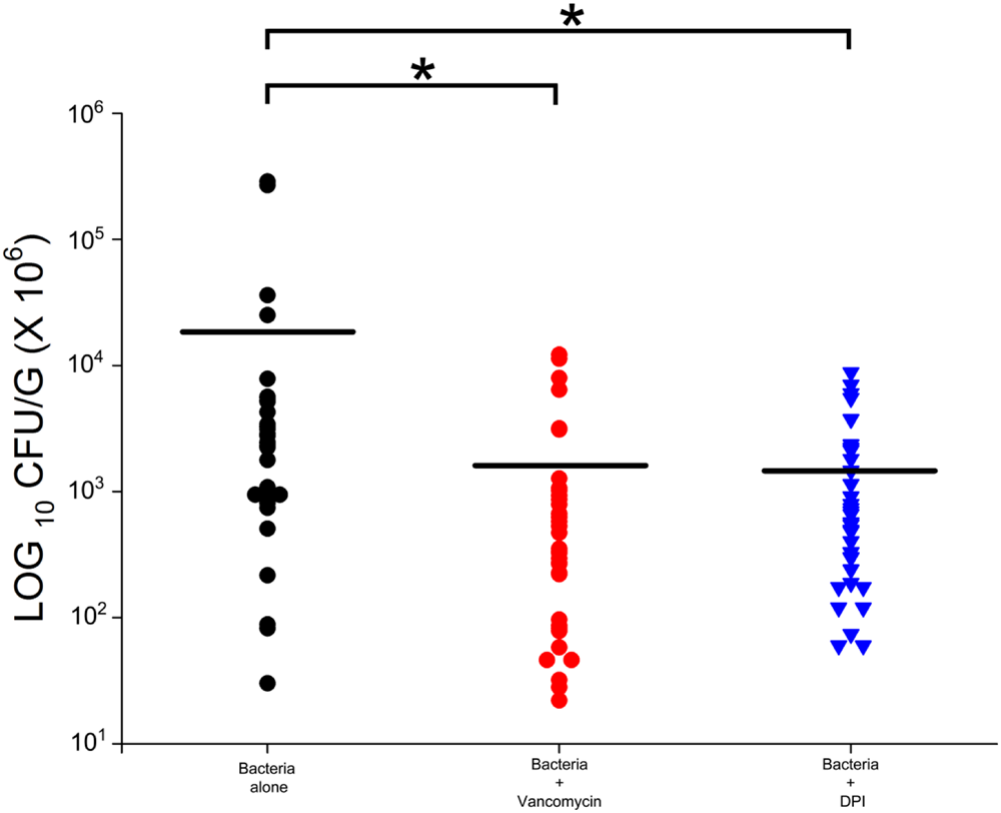
DPIC demonstrates efficacy in a murine neutropenic thigh infection model. The reduction in cfu/gm of tissue is plotted. The mice were treated with intraperitoneal doses at 3 h and 6 h of DPIC and vancomycin post-infection. P < 0.05, one-way ANOVA.

Since, the *in vitro* susceptibility of a pathogen to a drug can be markedly different from the *in vivo* susceptibility, we tested the efficacy of the DPIC against *S. aureus* infection in a murine neutropenic thigh infection model. The MTD of DPIC was determined to be 1 mg/Kg (data not shown). This model has been utilized extensively for evaluating the *in vivo* efficacy of several molecules against *S. aureus*^22,23^. Briefly, the thigh of neutropenic mice was infected with *S. aureus*, followed by two IP injections of DPIC at 1 mg/Kg while vancomycin was injected at 25 mg/Kg. Saline and vancomycin treated mice groups were used as a negative and positive control respectively. As seen in Fig. 3, treatment with DPIC significantly reduced mean bacterial counts in thigh compared to control group (P < 0.05), which is comparable to vancomycin. Vancomycin at 25 mg/Kg caused a reduction of ~1 log_10_ cfu while DPIC at 1 mg/Kg caused a reduction of ~1.2 log_10_ cfu in 24 h as compared to no-drug control. These results reveal that DPIC is as effective as vancomcyin in reducing the bacterial load in infected mice, that too at 1/25 dosage.

Due to the rapid emergence of drug resistant pathogens, managing infections that are otherwise treatable has become very challenging. A steep decline in the discovery of novel antibiotics has left us with very limited options to combat the increasing menace of AMR. Thus, the discovery of novel molecules with potentially new mechanisms of action is the need of the hour. We, in the current study, have identified a novel non-antibiotic compound DPIC that demonstrated potent bactericidal activity, against not only the laboratory strains but also against clinically isolated drug sensitive and drug-resistant strains of Mtb and *S. aureus*, at nano and micro-molar concentrations respectively. Finally, the efficacy of DPIC was validated in both an *in-vitro* Mtb macrophage infection and an *in-vivo S. aureus* murine neutropenic thigh *S. aureus* infection model. The current challenge with DPIC is its very narrow therapeutic index (TI) for *S. aureus* but it is not a significant concern for deployment of DPIC against TB. Improving upon the therapeutic index through designing of DPIC analogues is currently a major area of research in our group.

## Materials and Methods

### Drug library and Compounds

The LOPAC^®^1280 (Library of Pharmacologically Active Compounds, Sigma-Aldrich, USA) contained 1280 structurally diverse small molecules, including inhibitors of GPCR’s, kinases and FDA approved drugs, in a 96 well format as 10 mM stocks of drugs in DMSO. All the other chemicals were of the highest grade available.

### Bacterial strains and media

The mycobacterial strains were susceptible Mtb H_37_Rv ATCC 27294, INH-resistant Mtb H_37_Rv ATCC 35822, RIF-resistant H_37_Rv ATCC 35838, STR-resistant H_37_Rv ATCC 35820 and ETB-resistant H_37_Rv ATCC 35837. The other bacterial panel consisting of ESKAPE pathogens namely *Escherichia coli* (ATCC 25922), *Klebsiella pneumoniae* (BAA-1705), *Acinetobacter baumannii* (BAA-1605), *Pseudomonas aeruginosa* (ATCC 27853), *Staphylococcus aureus* (ATCC 29213) and *Enterococcus* sp. This panel was further expanded to include drug-resistant clinical *S. aureus* strains including resistant to Vancomycin and other clinically utilized antibiotics. These strains were procured from BEI/NARSA/ATCC (Biodefense and Emerging Infections Research Resources Repository/Network on Antimicrobial Resistance in *Staphylococcus aureus*/American Type Culture Collection, USA) and routinely cultivated on Mueller-Hinton Agar (MHA). Before starting the experiment, a single colony was picked from MHA plate, inoculated in Mueller-Hinton cation supplemented broth (MHB) and incubated overnight at 37 °C with shaking for 18–24 h to get the starter culture.

The mycobacterial strains were cultured in Middlebrook 7 H9 enriched (Difco, Becton, NJ, USA) media supplemented with 10% Oleic acid, Bovine Serum Albumin, Dextrose, 0.2% glycerol and 0.05% Tween-80 (OADC-Tween-80). Middlebrook 7H11 agar supplemented with 10% OADC plates used for bacterial cell enumeration by Colony Forming Unit (cfu) assay.

### Library Screening

LOPAC^®^1280 was screened for inhibition of mycobacterial growth. *Mycobacterium bovis* BCG was grown to 0.4 to 0.5 OD_600nm_ and diluted to an OD_600nm_ 0.005 in 7H9 enriched media. Outer perimeter of 96 well microtiterplate was filled with 200 µl sterile water to prevent dehydration in wells during incubation. 100 µl of diluted culture was transferred to the columns 2 to 11. 4 µl of 500 µm concentration of each compound from stock plates were transferred to the wells from column 2 to 11 using multi-channel pipetting to obtain a final concentration of 10 µm in each well. Media only, bacteria only and antibiotic controls in triplicate were established in each plate. Culture plates were sealed with parafilm M (Bemis, USA) and incubated at 37 °C for 5 days. On fifth day, 20 µl of Presto blue (Thermo scientific, USA) were added in each well. Fluorescence (excitation wavelength 560 nm and emission at 590 nm) readout was taken after incubating the plate for 24 hours using a synergy HT multimode reader^17^.

### Minimal inhibitory concentration assays

Antibacterial susceptibility testing was carried out utilizing broth microdilution assay according to CLSI guidelines. 10 mg/mL stock solutions of test and control compounds were prepared in DMSO and stored in −20 °C. Bacterial cultures were inoculated in appropriate media and OD_600_ of the cultures was measured, followed by dilution to achieve ~10^5^ CFU/mL. The compounds were tested from 64–0.5 mg/L in two-fold serial diluted fashion with 2.5 μL of each concentration added per well of a 96-well round bottom microtiter plate. Later, 97.5 μL of bacterial suspension was added to each well containing the test compound along with appropriate controls. Presto blue (Thermo Fisher, USA) resazurin -based dye was used for the visualized identification of active drugs. MIC of active compound was determined as lowest concentration of compound that inhibited visible growth after incubation period^24^. For each compound, MIC determinations were replicated three times using duplicate samples. The MIC plates were incubated at 37 °C for 18–24 h for (ESKAPE group) and 7 days for Mtb.

### Cell Cytotoxicity Assay

Cell toxicity was performed against Vero cell using the MTT assay. ~10^3^ cells/well were seeded in 96 well plate and incubated at 37 °C with an 5% CO_2_ atmosphere. After 24 h, compound was added ranging from 100–12.5 mg/L and incubated for 72 h at 37 °C with an 5% CO_2_ atmosphere. After the incubation was over, MTT was added at 5 mg/L in each well, incubated at 37 °C for further 4 hours, residual medium was discarded, 0.1 mL of DMSO was added to solubilise the formazan crystals and OD was taken at 540 nm for the calculation of CC_50_. CC_50_ is defined as the lowest concentration of compound which leads to a 50% reduction in cell viability. Doxorubicin was used as positive control and each experiment was repeated in triplicate.

### Bacterial time kill kinetics

The presence or absence of bactericidal activity was assessed by time-kill method. Mtb H_37_Rv ATCC 27294 was diluted to ~10^5^ cfu/mL, added to 96 well plate along with DPIC and appropriate controls at 1x and 5x MIC, followed by incubation at 37 °C for 7 days. For evaluating the reduction in CFU, 0.1 mL sample was removed at various time-points, serially 10-fold diluted in 0.9 mL phosphate buffer saline and 0.1 mL of the respective dilution was spread on Middlebrook 7H11 agar plate supplemented with OADC. The plates were incubated at 37 °C for 3–4 weeks and colonies were enumerated. Kill curves were constructed by counting the colonies from plates and plotting the CFU/mL of surviving bacteria at each time point in the presence and absence of compound. Each experiment was repeated three times in duplicate and the mean data is plotted﻿.

For *S. aureus*, the presence or absence of bactericidal activity was assessed by the time-kill method. Briefly, *S. aureus* ATCC 29213 bacteria were diluted ~10^5^ CFU/mL in MHB and treated with 1X and 10X of MIC of DPIC and vancomycin and incubated at 37 °C with shaking for 24 h. 100 µL samples were collected at the time intervals of 0 h, 1 h, 6 h and 24 h, serially diluted in PBS and plated on MHA followed by incubation at 37^0^C for 18–20 h. Kill curves were constructed by counting the colonies from plates and plotting the CFU/mL of surviving bacteria at each time point in the presence and absence of compound. Each experiment was repeated three times in duplicate and the mean data is plotted.

### Determination of *ex-vivo* efficacy in murine Bone marrow derived macrophages (BMDM)

The BMDM were harvested from C57BL/6 mice and matured in DMEM media supplemented with 10% FBS and L929 cell line soup. After maturation, the cells were infected with Mtb H_37_Rv ATCC 27294 at 1:1 MOI. After 4 hours of infection, the cells were washed with PBS three times to remove the extracellular bacteria and defined concentration of various compounds and controls was added. After 3 days, the media was replaced with fresh drug-containing media. At sixth day post infection, cells were lysed using 0.01% triton X-100 and plated on 7H11 agar plates supplemented with 10% OADC supplement for enumeration of bacterial count.

### Synergy of DPIC with front-line anti-mycobacterial drugs

Determination of interaction with RIF and INH was tested by the checkerboard method as per CLSI guidelines. Serial two-fold dilutions of each drug were freshly prepared prior to testing. DPIC was two-fold diluted along the ordinate ranging from 0.03–0.0009 mg/L (5 dilutions) while the antibiotics were serially diluted along the abscissa ranging from 0.0625–0.0019 mg/L for RIF and INH and 0.125–0.0039 mg/L (5 dilutions) in 96 well microtiter plate. 95 µl of ~10^5^ CFU/mL was added to each well and plates were incubated at 37^0^C for 7 days. After the incubation period was over, the ΣFICs (fractional inhibitory concentrations) were calculated as follows: ΣFIC = FIC A + FIC B, where FIC A is the MIC of drug A in the combination/MIC of drug A alone and FIC B is the MIC of drug B in the combination/MIC of drug B alone. The combination is considered synergistic when the ΣFIC is ≤0.5, indifferent when the ΣFIC is >0.5 to 4, and antagonistic when the ΣFIC is >4^25^.

### Animal experiment

Animal experiments were performed on six-eight week old Balb/c mice procured from National Laboratory Animal Facility of CSIR-Central Drug Research Institute, Lucknow. The experimental protocols were reviewed and approved by the Institutional Animal Ethics Committee of CSIR-Central Drug Research Institute, Lucknow. Animal experiments were performed in accordance with the guidelines provided by the Committee for the Purpose of Control and Supervision of Experiments on Animals (CPCSEA, Govt. of India).

### Murine neutropenic thigh infection model

For *in vivo* antimicrobial activity evaluation of DPIC, female BALB/c mice weighing approximately 18–20 gm were used throughout the study. Mice were rendered neutropenic by a series of cyclophosphamide injections given intraperitoneally (IP) 1 day and 1 h before infection. This was followed by injection of *S. aureus* ATCC 29213 in the right thigh of mice to establish infection. After 3 h post infection, DPIC and vancomycin at 1 mg/Kg and 25 mg/Kg of body weight respectively, were injected IP into mice twice at an interval of 3 h between injections. Control animals were administered saline in the same volume and frequency as those receiving treatment. After 24 h, the mice were sacrificed, thigh tissue were collected from the animal and weighed. Collected tissue was homogenized in 5 mL of saline, serially diluted followed by plating on MHA plates for CFU determination. After incubation for 18–24 h at 37 °C, the CFU were enumerated. Each experiment was repeated three times in duplicate and the mean data is plotted.

### Statistical analysis

Statistical analysis was performed using GraphPad Prism 6.0 software (GraphPad Software, La Jolla, CA, USA). Comparison between three or more groups was analyzed using one-way ANOVA, with post-hoc Tukey’s multiple comparisons test. P-values of < 0.05 were considered to be significant.

## Electronic supplementary material

Supplementary info
