# Author Correction: Diphenyleneiodonium chloride (DPIC) displays broad-spectrum bactericidal activity

**DOI:** 10.1038/s41598-018-22720-z

**Published:** 2018-04-12

**Authors:** Manitosh Pandey, Alok Kumar Singh, Ritesh Thakare, Sakshi Talwar, Pratiksha Karaulia, Arunava Dasgupta, Sidharth Chopra, Amit Kumar Pandey

**Affiliations:** 1 Mycobacterial Pathogenesis Laboratory, Vaccine and Infectious Disease Research Centre (VIDRC), Translational Health Science and Technology Institute (THSTI), NCR Biotech Science Cluster, Faridabad-Gurugram Expressway, Faridabad, 121001 Haryana India; 2 Division of Microbiology, CSIR-Central Drug Research Institute, Sector-10, Janakipuram Extension, Sitapur Road, Lucknow, 226031 Uttar Pradesh India

Correction to: *Scientific Reports* 10.1038/s41598-017-11575-5, published online 14 September 2017

The original version of this Article contained errors.

The rows in Table 1 were misaligned and this has now been corrected.

In addition, the Acknowledgements section in this Article was incomplete.

“This study was supported by the Ramalingaswami Fellowship (BT/RLF/Re-entry/45/2010) and RGYI grant (BT/PR6556/GBD/27/459/2012) from Department of Biotechnology, Govt. of India to A.K.P. S.T. is funded by research fellowship from ICMR while A.K.S and R.T thank CSIR for their fellowship. Intra-mural funding by THSTI and CSIR-CDRI is acknowledged. We thank the Director, ICGEB, and TACF staff, ICGEB, New Delhi for use of the BSL-3 facility. The following reagents were provided by the Network on Antimicrobial Resistance in Staphylococcus aureus (NARSA) for distribution by BEI Resources, NIAID, NIH: NR 119, NR 100, NR 10129, NR 10198, NR 10192, NR 10191, NR 10193, NR 10186, NR 10194, VRS1, VRS4 and VRS12.”

now reads:

“This study was supported by the Ramalingaswami Fellowship (BT/RLF/Re-entry/45/2010) and RGYI grant (BT/PR6556/GBD/27/459/2012) from Department of Biotechnology, Govt. of India to A.K.P. S.T. is funded by research fellowship from ICMR while A.K.S and R.T thank CSIR for their fellowship. Intra-mural funding by THSTI and CSIR-CDRI is acknowledged. We thank the Director, ICGEB, and TACF staff, ICGEB, New Delhi for use of the BSL-3 facility. The following reagents were provided by the Network on Antimicrobial Resistance in Staphylococcus aureus (NARSA) for distribution by BEI Resources, NIAID, NIH: NR 119, NR 100, NR 10129, NR 10198, NR 10192, NR 10191, NR 10193, NR 10186, NR 10194, VRS1, VRS4 and VRS12. This manuscript bears CDRI communication number 9556.”

Finally, this Article contained an error in the Author Information section where:

“Manitosh Pandey and Amit Kumar Pandey contributed equally to this work.”

now reads:

“Manitosh Pandey and Alok Kumar Singh contributed equally to this work.”

These errors have now been corrected in the PDF and HTML versions of the Article and in the accompanying Supplementary Materials file.

