# Supplementary material for: Diphenyleneiodonium chloride (DPIC) displays broad-spectrum bactericidal activity
Source: Sci Rep. 2017 Sep 14;7:11521. doi: 10.1038/s41598-017-11575-5 (PMC5599662; doi:10.1038/s41598-017-11575-5)
Supplement: Supplementary file 1 — Supplementary info [file 41598_2017_11575_MOESM1_ESM.pdf]

Title: Diphenyleneiodonium chloride (DPIC) displays broad-spectrum bactericidal activity

Manitosh Pandey[1*], Alok Kumar Singh[2*], Ritesh Thakare[2], Sakshi Talwar[1], Pratiksha Karaulia[2], Arunava Dasgupta[2], Sidharth Chopra[2#] and Amit Kumar Pandey[1#]

[1]Mycobacterial pathogenesis laboratory, Vaccine and Infectious Disease Research Centre (VIDRC), Translational Health Science and Technology Institute (THSTI), Faridabad 121001, Haryana, India; [2] Division of Microbiology, CSIR-Central Drug Research Institute, Lucknow-226031, Uttar Pradesh, India.

[*]contributed equally to this study
[#]Correspondence and requests for materials should be addressed to A.K.P. (email: amitpandey@thsti.res.in) , S.C. (email: skchopra007@gmail.com)

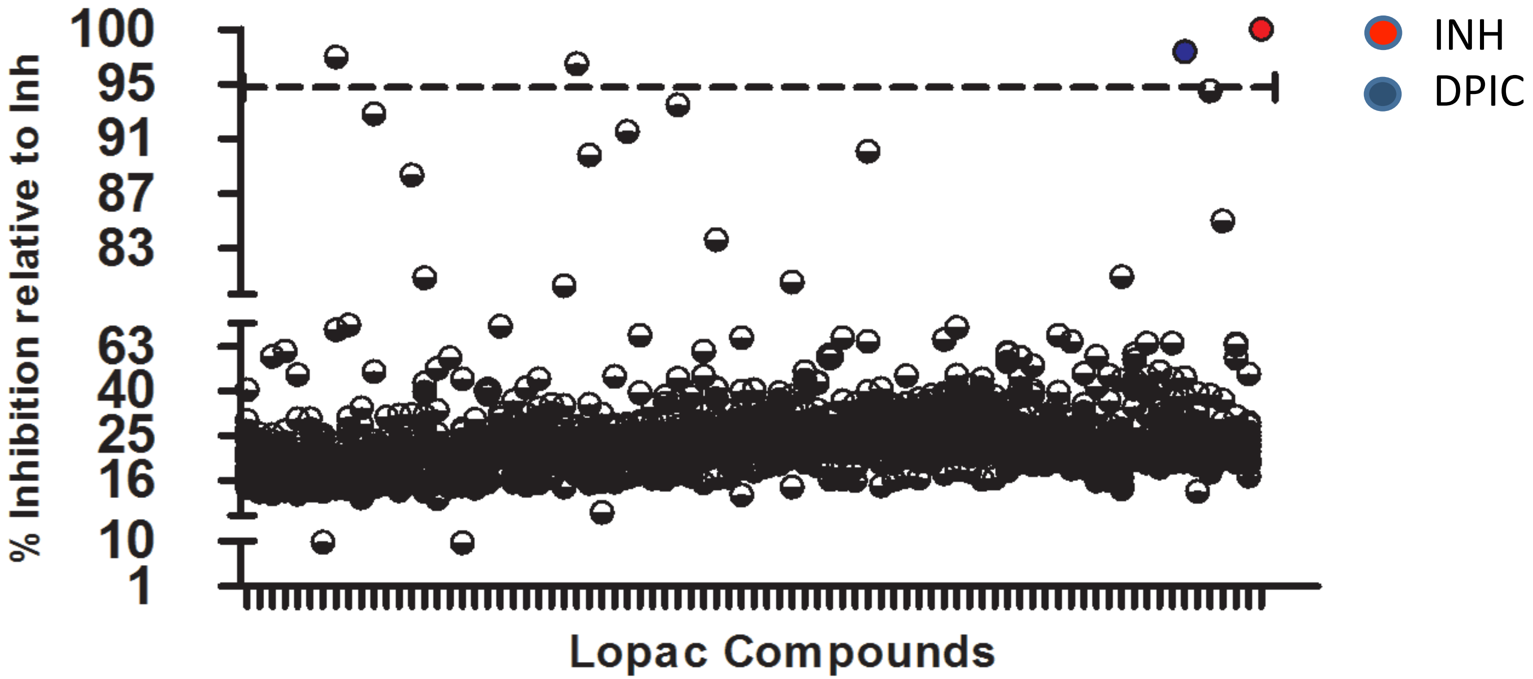

Supplementary Fig. 1. Resazurin dye based high-throughput screening of LOPAC library. LOPAC library consisting of 1280 drugs were screened against *M. bovis* BCG in 7H9 enriched media. The drugs were screened at 10µM concentration. First-line anti-tuberculosis drug Isoniazid (INH) was used as a positive control

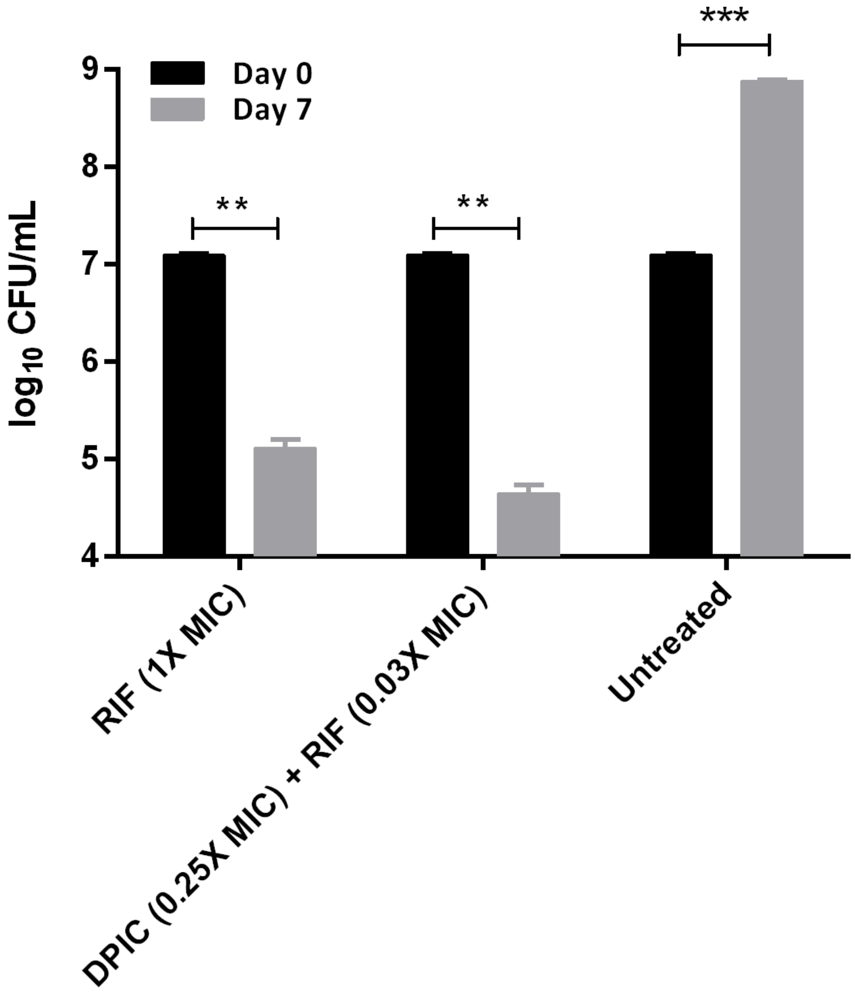

Supplementary Fig. 2. DPIC synergises the anti microbicidal activity of Rifamycin (RIF). Killing of Mtb in the presence of 1X MIC (0.03 mg/L) of RIF and in combination of RIF and DPIC at 0.03X and 0.25X MIC respectively. Bacterial enumeration was done at day 0 and day 7 on 7H11+OADC plates. *P<0.05, **P<0.001, unpaired two tailed t-test.

**Drug**	MIC (mg/L)	
	**pH 7.2**	**pH 5.5**
Pyrazinamide	>100	25
DPIC	0.03	0.03

Supplementary Table. 1. Anti-microbial property of DPIC is independent of pH
